# Discovery of a deep-sea coral garden of *Crypthelia vascomarquesi* (Hydrozoa: Hydroidolina) in the Menez Gwen marine protected area (Mid-Atlantic ridge)

**DOI:** 10.1007/s12526-026-01624-6

**Published:** 2026-03-10

**Authors:** Íris Sampaio, José Nuno Gomes-Pereira, Ana Colaço, André Freiwald, Fernando Tempera

**Affiliations:** 1 Abteilung Meeresforschung, Senckenberg Am Meer, Südstrand 40, 26382 Wilhelmshaven, Germany; 2 CIIMAR/CIIMAR LA, Interdisciplinary Centre of Marine and Environmental Research, University of Porto, Terminal de Cruzeiros Do Porto de Leixões, Avenida General Norton de Matos, S/N, 4450-208 Matosinhos, Portugal; 3 IMAR — University of the Azores, Rua Prof. Dr. Frederico Machado 4, 9901-862 Horta, Açores Portugal; 4 Naturalist — Science & Tourism, Atlantic Naturalist Association, Rua da Ladeira 2, 9900-029 Horta, Açores Portugal; 5 University of the Azores, OKEANOS, Rua Professor Doutor Frederico Machado 4, 9901-862 Horta, Açores Portugal; 6 Unité Biologie des Organismes et des Ecosystèmes Aquatiques – BOREA (équipe Aquatrends), Sorbonne Université, MNHN, UA, CNRS, IRD, Paris, France

**Keywords:** Azores, EUNIS habitat classification system, Hydrocoral, North Atlantic, Stylasteridae, Vulnerable marine ecosystem

## Abstract

*Crypthelia* Milne-Edwards & Haime, 1849 is the most diverse genus of lace corals inhabiting the Azores. During the 2012 DEEPFUN cruise, the first coral garden structured by this genus was recorded in the Menez Gwen Marine Protected Area (MPA). Identification of the species was based on morphology of specimens collected in situ and characterized using scanning electron microscopy (SEM). Imagery acquired by the remotely operated vehicle (ROV) Victor 6000 was used to document the species density as well as seabed geomorphology. The stylasterid *Crypthelia vascomarquesi* Zibrowius & Cairns, 1992 is described for the first time based on entire specimens, including female representatives. To date, this is the only *Crypthelia* species with female efferent pores located on dactylostomes, adding a new category to the feminine ampullar formula developed for the genus. The dense coral garden formed by *C. vascomarquesi* was recorded between 832 and 856 m depth at 37°48.896′N; 31°33.774′W. This vulnerable marine ecosystem (VME) represents a rare record of fragile and highly endemic corals that reinforces the value of natural heritage protected within a mid-Atlantic ridge MPA. We suggest that the *C. vascomarquesi* coral garden is included at level 5 of the deep-sea section of the European habitat classification system (EUNIS), as a means to emphasize its conservation value and the monitoring of MPAs.

## Introduction

The first deep-sea explorations of Northeast Atlantic Ocean date back to the late 19^th^ century, when a series of pioneering scientific expeditions sampled the first pristine and virtually unknown grounds on and off the European margins, including the Azores (Duncan 1873; Murray et al. 1912). More than a century later, deep-sea research in the region has been boosted by the recognition of the ever-increasing impacts of human activities produced in deep-sea ecosystems (Sampaio et al. 2012; Morato et al. 2022). With concerns about existing fishing activities and prospective activities like deep-sea mining extending well beyond continental margins, research has focused on the description and mapping of deep-sea biotopes, which remain poorly known despite their vulnerability to human activities (Jones et al. 2025). The Azores has proven its hotspot quality for deep-sea organisms like corals, which have shown a remarkable diversity and widespread distribution in the topographically rich deep-sea areas that surround the archipelago (Braga-Henriques et al. 2013; Sampaio et al. 2019). A systematical classification of the coral-structured biotopes in the Northeast Atlantic along with an illustrated catalogue of the imagery of Azorean deep-sea facies (Tempera et al. 2013; Davies et al. 2017) has revealed biotopes frequently composed by multispecific coral gardens, generally dominated by octocorals (Sampaio et al. 2019). While the deep-sea reefs of northern latitudes are characterized by scleractinians, only a few examples have been found on the Mid-Atlantic Ridge and some island and seamount slopes (Hovland et al. 2002). However, descriptions of the composition of the Azorean deep-sea biotopes remain scarce (Matos et al. 2014; Gomes-Pereira and Tempera 2016; Morato et al. 2021).

Stylasterids are a particular group of hydrozoans that exhibit hard calcareous skeletons (Cairns 1986). Lace corals are less studied in comparison with other groups of corals and, if we consider their high percentage of endemism, they have the lowest dispersal ability of all (Cairns 1986). The endemic and emblematic *Errina dabneyi* (Pourtalès, 1871) is common by-catch of the local longline fishery of the Azores and it was observed by submersible between 200 and 500 m depth (Wisshak et al. 2009; Sampaio et al. 2012). This stylasterid species was found forming coral gardens in association with *Viminella flagellum* (Johnson, 1863) and large leptothecate hydroids on mixed seabed at 200 m depth, but also in association with “lithistid” sponges’ on exposed rocky edges of seamounts and island slopes between 350 and 400 m depth (Tempera et al. 2013).

*E. dabneyi* is the most abundant habitat-forming species within the nine stylasterids reported for the Azores. Yet, the genus *Crypthelia* Milne-Edwards & Haime, 1849 is the most diverse genus in the region. It is represented by four species, notably: *Crypthelia affinis* Moseley, 1879, *C. medioatlantica* Zibrowius & Cairns, 1992, *C. vascomarquesi* Zibrowius & Cairns, 1992, and *C. tenuiseptata* Cairns, 1986 (Zibrowius and Cairns 1992).

In 2012, the DEEPFUN cruise (Biodiversity and functioning of the deep-sea hydrothermal field Menez Gwen—a contribution for management policies) conducted onboard the RV *Thalassa* has targeted a hydrothermal vent field and neighboring grounds, leading to the discovery of a stylasterid coral garden. In this study, we describe the first monospecific coral garden dominated by a lace coral for the Azores. Herein, the stylasterid species of *Crypthelia* forming the coral garden is described considering its associated fauna and  geomorphological context interpreted from high-resolution bathymetry. Knowledge on deep-sea habitats formed by endemic species enhances its conservation importance and is fundamental for proposals with the representativeness, balance, and ecological coherence of the Natura 2000 and OSPAR MPA networks.

## Materials and methods

### Study area

The Menez Gwen Hills are located on the Mid-Atlantic Ridge (MAR) in a segment positioned between the Princess Alice Bank Offset at 38°N, to the north, and the Pico Offset at 37.5°N, to the south (Klischies et al. 2019). The segment is dominated by a circular volcano emplaced in its central part exhibiting a 700 m height and a 17 km diameter. This edifice is split into two mirror halves by a 2 km width and 9 km length axial graben striking across the volcano along a NNE-SSW direction. The throw of the subsided central block ranges between 300 and 400 m (Fouquet et al. 1995; Marcon et al. 2013).

Since the discovery of its active hydrothermal field in 1992 during the French cruise DIVA1, this site has received considerable attention, notably from French, German, Portuguese, and North-American scientific cruises. With a convenient half-day transit time from a major harbor, this is also the shallowest known bathyal hydrothermal area within the Azores EEZ, with depths ranging from 847 to 871 m (Ondréas et al. 1997; Marques et al. 2009; Borowski et al. 2010; Marcon et al. 2013).

Hydrothermal activity occurs over an area of approximately 200 m^2^ near the top of a young 120 m height volcano located at the northern end of the graben between 37°50.8′–37°51.6′N latitude and 31°30′–31°31.8′W longitude (Fouquet et al. 1994, 1995). A smaller hydrothermal field named Bubbylon was discovered in 2010 at ca. 1000 m water depth during the Meteor Cruise M82/3. The field lies 5 km to the SSW of main Menez Gwen site at 37°47.9′N latitude and 31°32.0′W longitude. Venting occurs through the talus material piled up at the base of the east-facing fault scarp of the axial graben (Dubilier and the M82/3 scientific party 2012; Schiellerup et al. 2021).

Temperatures of the fluids emitted by the Menez Gwen vents range between 265 and 281 °C and pH levels are between 4.2 and 4.8. The fluids are enriched with methane (CH_4_) but hydrogen sulfide (H_2_S) contents are low compared to other hydrothermal fields (< 2 mmol/kg) (Santos et al. 2003). Fluid sampled on the Bubbylon field exhibited slightly lower temperatures (253–254 °C) and pH levels that ranged from 3.9 to 5.3, containing large amounts of both methane and hydrogen sulfide (Dubilier and the M82/3 scientific party 2012; Schiellerup et al. 2021).

**Fig. 1:**
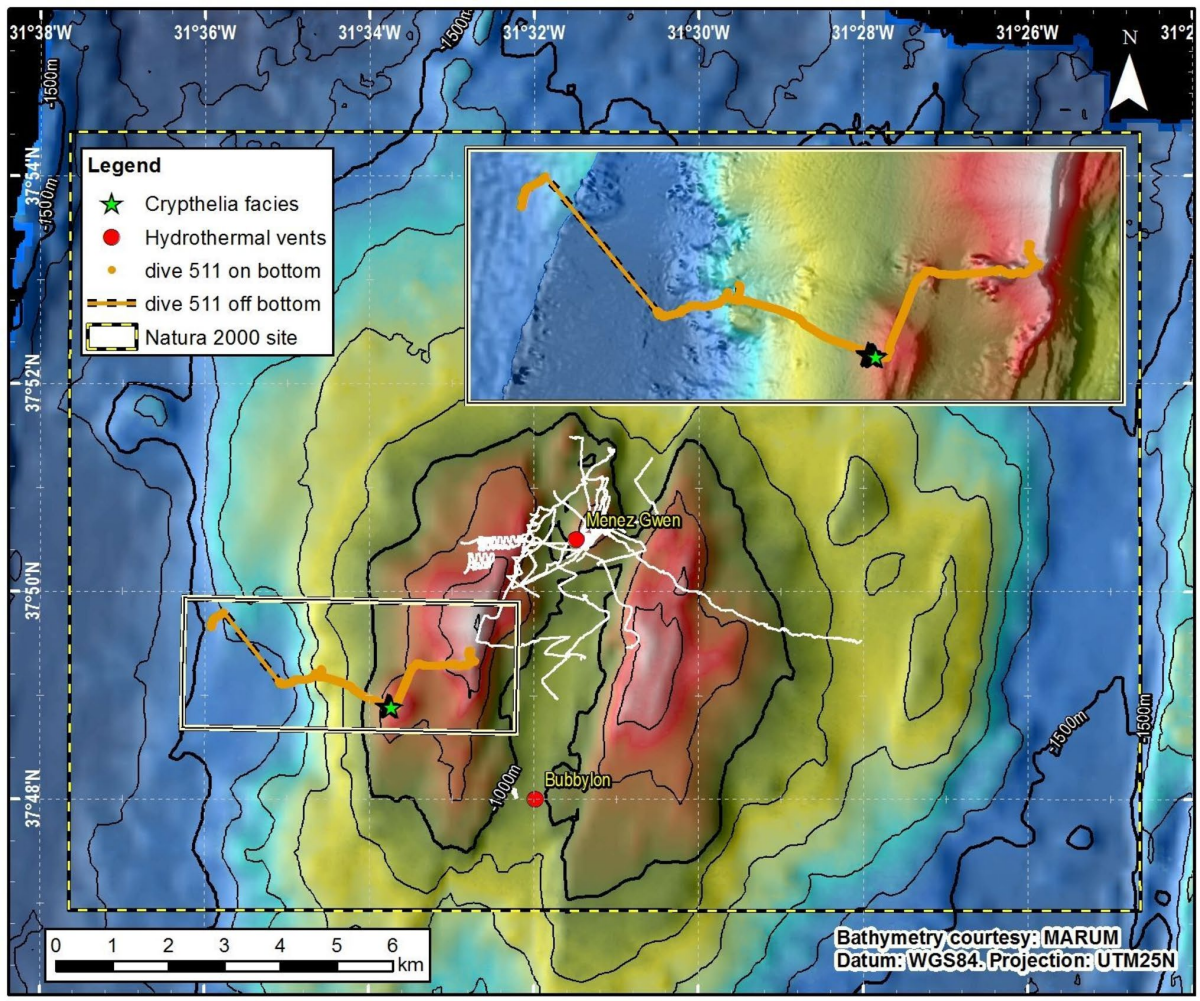
Bathymetry of the Menez Gwen Marine Protected Area. Tracks from exploration dives conducted previous to the DEEPFUN cruise by French, German and Portuguese scientific cruises are shown as white lines. The track of the exploration dive (#511) leading to the discovery of the *Crypthelia* facies is shown in orange. The locations of both the Menez Gwen and Bubbylon hydrothermal fields are shown as red dots. The inset details and topographical features visited along the track with the facies location being indicated by a star. 3-D close-up of the facies location. Bathymetry credits: Dubilier (2013b). Dive track credits: Ifremer, Marum and IMAR/DOP-UAz.

As a result of the conservation importance of its hydrothermal biological communities and coral communities, the Menez Gwen Hills were classified in 2007 as an OSPAR Marine Protected Area. One year later it was designated as a marine Natura 2000 Site of Community Importance, and in 2011 it integrated the Marine Park of the Azores as an IUCN category Ib MPA (Wilderness Area) (Probert et al. 2007; Calado et al. 2011; Fig. 1).

The seabed directly influenced by the hydrothermal vent is dominated by mussel beds and shrimp aggregations (Desbruyères et al. 2001) and do not seem to represent a favorable environment for corals. Yet, away from direct hydrothermal influence an abundance of hard seafloor gardens formed by both hard and soft corals have been reported (Tempera et al. 2013). Large patches of dead *Madrepora oculata* Linnaeus, 1758 framework have also been observed away from the vents, but the conditions that sustained them seem to have declined long ago, as most of the framework is covered by Fe-Mn deposits and only a few living coral heads persist.

### Experimental design and data acquisition

A review of the navigation tracks of the dives performed by manned submarines and remote operated vehicles (ROVs) during Ifremer, MARUM, and IMAR expeditions suggested a strong bias of the scientific effort towards the Menez Gwen active hydrothermal vent field which sits on a small axial elevation. Among all dives inventoried in 2011 for the area (ca. 70), only 14 explored sizeable areas outside the hydrothermal field. Their depths ranged between 1125 m and the shallowest point of the Menez Gwen Hills at 689 m depth. In the whole explored area, there were multiple bathyal habitats of interest, including coral gardens, dead scleractinian reefs and xenophyophore fields (Tempera et al. 2013).

Furthermore, the compilation of past dives highlighted the unexplored condition of the western flank of the volcano, where high resolution bathymetry has revealed a suite of geomorphological features. A transect was therefore planned to explore the seafloor diversity and depth-wise succession of biological facies along the whole western Menez Gwen hill flank.

### Collection of bathymetry and imagery

The DEEPFUN cruise took place on board RV *Thalassa* during the summer of 2012*.* Video and still imagery was collected by ROV Victor 6000 on the 25^th^ July over a transect of the outer flank of the western Menez Gwen hill. The ROV Victor 6000 was equipped with two ultra-high-resolution cameras, a high definition still camera associated with flashes providing good quality imagery of the observed animals. Detailed ROV positioning was provided by an underwater positioning system which was instrumental in locating all seafloor observations.

### ROV video annotation

Seabed video footage was annotated using a customized interface on software COVER (Customizable Observation Video Image Recorder, ©IFREMER) focusing on describing the main seabed characteristics and compiling a list of visually identifiable taxa (Guillaumont 2011).

Colony density was estimated from a series of 15 photographs taken over the facies by the downward-facing camera #16. Density estimates did not differ significantly among four independent observers (ANOVA, *p* = 0.812), indicating consistent and coherent measurements.

Parameters used for the areal coverage calculation, including altitude and zoom level, were embedded in the frames themselves. Prior to annotation, the photos were corrected for pixel interlacing, lack of contrast and vignetting. The ImageJ 1.54p deinterlace plugin and the GIMP 2.10.38 tools for automated white balancing and shadows/highlights adjustment where used, enhancing the detectability of colonies on the image periphery and in overexposed areas.

ImageJ *Multipoint* tool was used to count the number of colonies present in each photograph. Maximalist criteria were applied, with all colonies being counted irrespective of (i) their color (notably, white living colonies and possibly dead yellowish skeletons) or (ii) their angle relative to the camera (notably fixed colonies top views as well as possibly broken colonies planar views).

The total area represented in each photo was computed using a spreadsheet shared by Ifremer which permits, among other operations, estimating the areal coverage of individual frames taking account ROV altitude and zoom level.

Colony density was estimated by dividing the colony counts of each photo by the planar area it represented.

### Sampling and morphological analyses

During dive 511, eleven colonies and nine parts of hydrocoral colonies were collected using the ROV bioboxes. Upon arrival to the surface, the samples were transferred to the vessel’s wet lab and photographs were taken with a scale. Multiple subsamples were preserved using distinct methods and media (− 80 °C freezing, ethanol 96%, 10% formaldehyde, and air-drying).

In the lab, selected sub-samples were sputter-coated with gold and photographed with a Tescan VEGA3 XMU scanning electron microscope at Senckenberg am Meer in Wilhelmshaven for detailed morphological analysis. Anatomical measures were taken with Image J 1.49 based on scaled images of specimens and of their microscopic structures. Identification was based on key taxonomical characters such as coenosteum, nematopores, cyclosystem, cyclosystem lid, dactylopores per cyclosystem, pseudosepta, and ampullar formula. Diagnostic features presented in the catalogues of Zibrowius and Cairns (1992) and Cairns (1986, 2011, 2015) served as the main references for the region of the Azores.

## Results

**Fig. 2:**
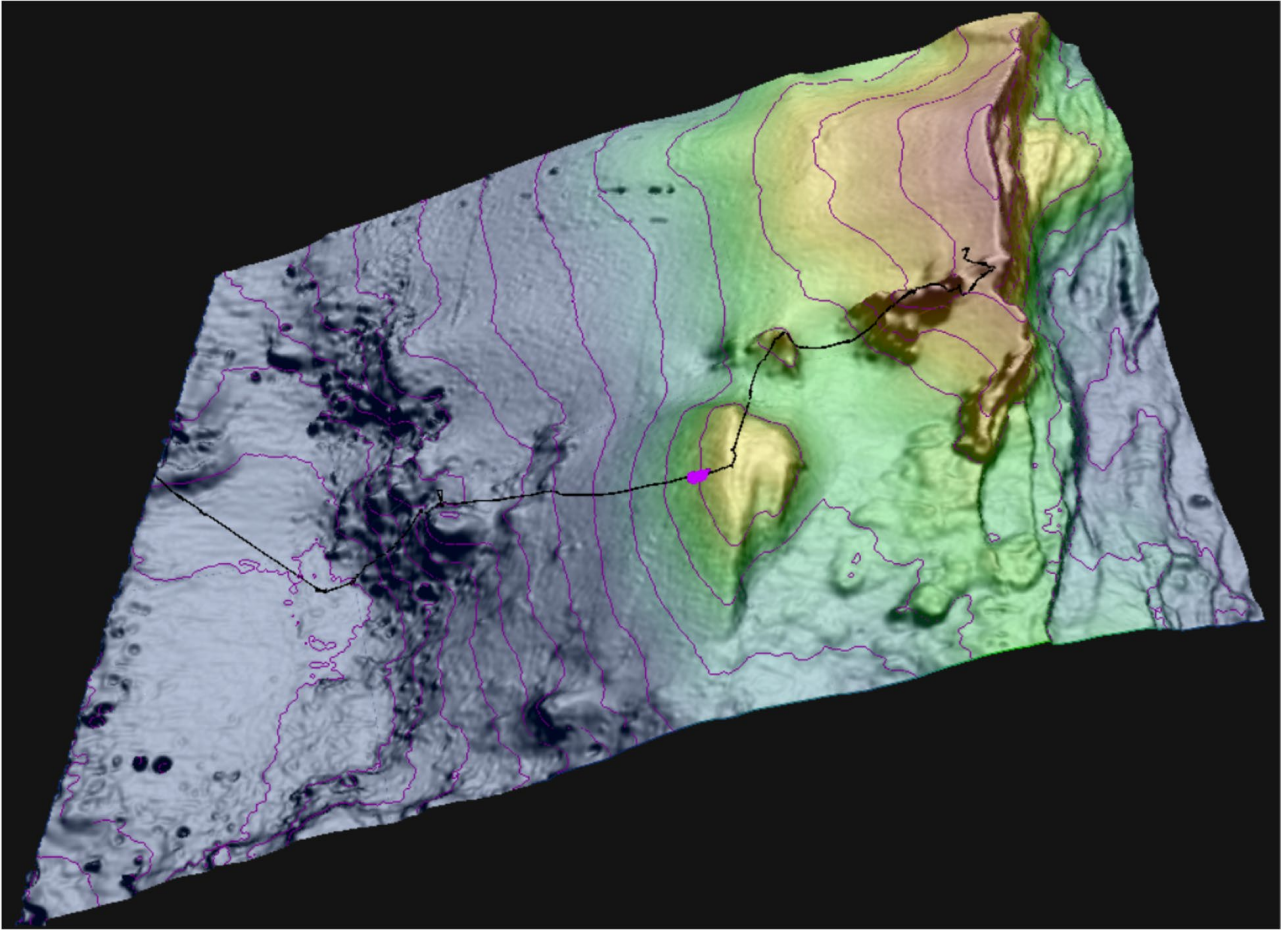
3-D perspective of the western Menez Gwen Hill flank showing the dive 511 track (black line) and the location of the *Crypthelia vascomarquesi* facies (purple patch). The east-facing fault of the axial graben is on the right. Bathymetry credits: Dubilier (2013b)

**Fig. 3:**
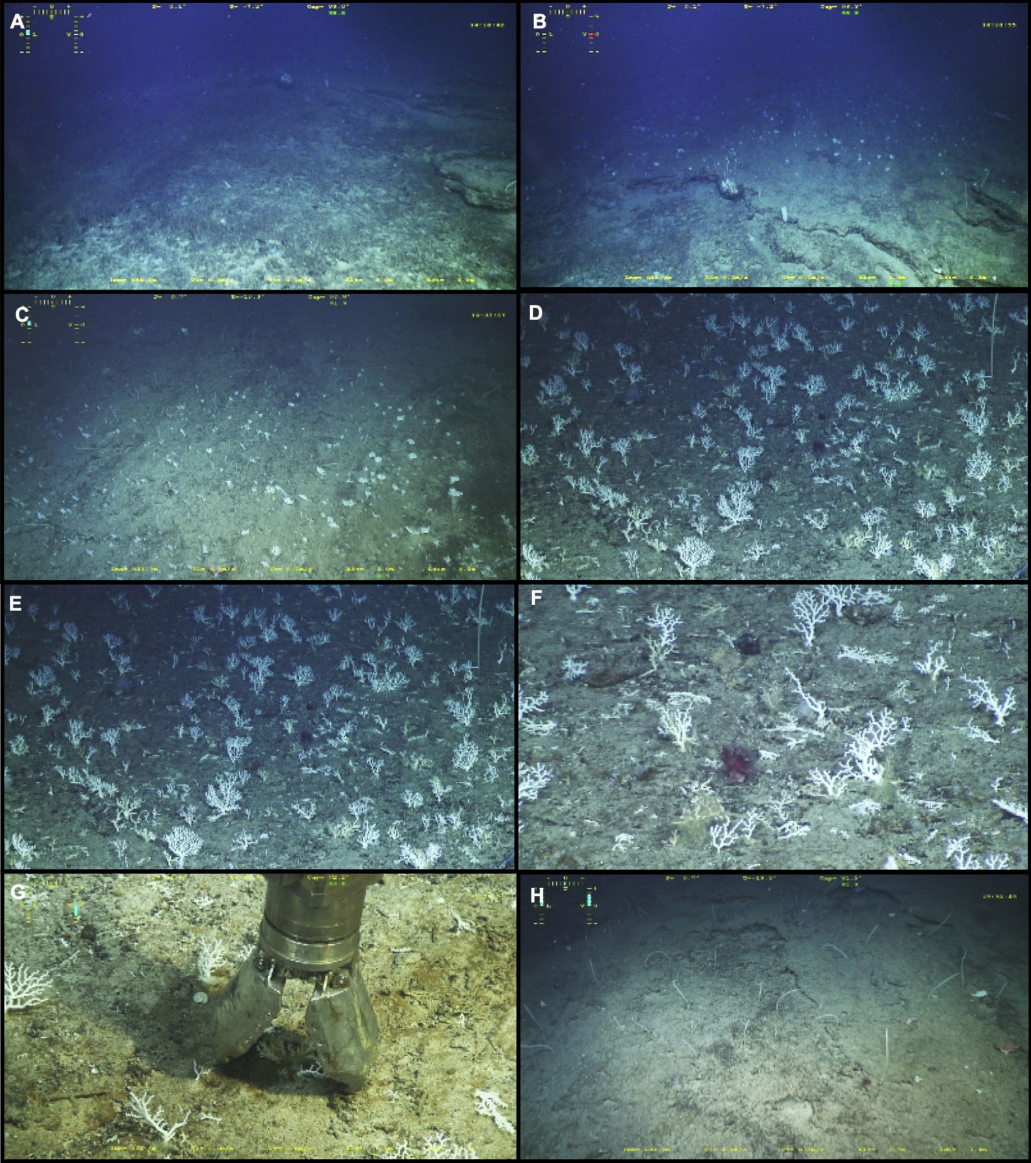
Seafloor imagery; **A** Slump scar with displaced block on the right and headwall in the back; **B** lower end of the *Crypthelia* facies which starts on a sector of unbroken consolidated crust located upslope from the thin slump headwall visible on the image; **C** and **D** dense *Crypthelia* at progressive close-ups of the seafloor; **E** upper end of *Crypthelia* facies, transitioning into a *Narella versluysi* facies; **F** Close-up of *Crypthelia* with cf. *Anthomastus* sp.; **G** collection of *Crypthelia* specimens with the ROV Victor 6000 grabber and; **H** facies of *Narella versluysi* (Hickson, 1909) upslope from the *Crypthelia* facies

A Ffacies largely dominated by *Crypthelia vascomarquesi* Zibrowius & Cairns, 1992 was located during the first extensive exploration of the outer flank of the western Menez Gwen hill, approximately 5 km to the SW of the Menez Gwen hydrothermal field and 3 km to the WNW of the Bubbylon vents. The facies extended 73 m in planar distance between its lower limit at 856 m depth and its upper limit at 832 m depth. It lay on the flank of a small parasitic elevation dipping between 16 and 21°, that we herein name Crypt’Hill given its association with the *Crypthelia* facies (Figs. 1, 2, and 3a–f). Dense *Crypthelia* stopped 70 m short in planar distance of the elevation summit, which is noticeably flatter on the digital terrain model and dips between 0 and 9°. No along-slope exploration of the facies was performed preventing any appraisal of its horizontal extent.

### Geomorphological context

The whole MPA has been thoroughly surveyed using an EM710 multibeam sonar (Dubilier, 2013a) resulting in a 5 m resolution bathymetry grid. With the exception of a few bulbous rocky outcrops, likely made up of pillow lavas, the west-facing slope at stake is fairly smooth and exhibits a low uniform backscatter (Figs. 1, and 2). This smoothness, which characterizes the volcano´s off-axis flanks, is attributed to a pyroclast and volcanic ejecta coating produced by explosive volcanism (Ondréas et al. 1997; Fouquet et al. 1998; Parson et al. 2000). Seabed imagery confirmed the prevalence of smooth surfaces dominated by consolidated sediments covered by patchy veneers of unconsolidated sediments (Fig. 3).

An analysis of Crypt’Hill bathymetry at 5 m resolution suggests that the substrate underlying the facies exhibits densely packed gentle along-slope undulations. These bedforms dip between 16 and 21°, resembling shallow downward creeping sediment waves. Although in situ imagery shows a thin mass wasting scar immediately below the facies, no flank indentation is resolved by the bathymetric grid suggesting its indentation is below the grid resolution.

### Macroscopic description of the facies

Dive 511 of DEEPFUN cruise started over a bathyal sedimentary ground on the base of the Hills and proceeded towards the summit of the western Menez Gwen Hill, traversing a series of elevations.

Coral facies largely dominated by the species *Crypthelia vascomarquesi* starts at 832 m depth with a bottom composed by unbroken sediment crust, upslope of the slump headwall (Figs. 1, 2, and 3). Specimens were observed on the western flank of the Menez Gwen Hills (Fig. 3a–g). The upper end of this monospecific coral garden largely dominated by the lace coral corresponds to the transition towards a multispecific coral garden including the primnoid gorgonian *Narella versluysi* (Hickson, 1909) and a diversity of other biota such as other coral species, sponges, crustaceans, and unidentified species, such as cf. *Anthomastus* sp. (Table 1, Fig. 3 e, f). Within-facies colony density ranged between 1.5 and 70.6 colonies per m^2^ with the lowest numbers corresponding to images where extensive parts of the imaged seabed were coated by more or less coarse sediment and coral rubble, which likely represented unsuitable substrate for the species.

**Table 1:** Marine invertebrates and fishes observed at the coral garden structured by *Crypthelia vascomarquesi* Zibrowius & Cairns, 1992

Phylum	Subphylum	Class	Order	Family	Species	Notes on morphology or ID
Cnidaria	Medusozoa	Hydrozoa	Anthoathecata	Stylasteridae	cf. *Lepidopora* sp.	
	Anthozoa	Hexacorallia	Antipatharia	Leiopathidae	*Leiopathes* sp.	
			Antipatharia			Unidentified black coral
			Scleractinia	Caryophylliidae	cf. *Desmophyllum*	
					cf. *Caryophyllia*	
		Octocorallia	Scleralcyonacea	Primnoidae	*Narella versluysi* (Hickson, 1909)	
				Coralliidae	*Hemicorallium tricolor* (Johnson, 1899)	
					cf. *Anthomastus* sp.	
				Keratoisididae	*Acanella* sp.	
						Red gorgonian
						Short orange bushy coral
Porifera		Hexactinellida	Lyssacinosida	Euplectellidae	cf. *Euplectella* sp.	
						Tubular white sponge > 10 cm
			Sceptrulophora	Aphrocallistidae	cf. *Aphrocallistes beatrix* Gray, 1858	
				cf. Farreidae		
						Yellow encrusting sponge < 10 cm
						Encrusting sponge
						gray sponge
Arthropoda	Crustacea	Malacostraca	Decapoda	Geryonidae	*Chaceon affinis* (A. Milne-Edwards & Bouvier, 1894)	
				Aristeidae	*Aristaeopsis edwardsiana* (Johnson, 1868)	
				Polybiidae	*Bathynectes maravigna* (Prestandrea, 1839)	
				Galatheidae		
						Natantia
Mollusca		Gastropoda	Trochida	Trochidae		White gastropod
Chordata	Vertebrata	Elasmobranchii	Squaliformes	Centroporidae	*Deania* sp.	
		Teleostei	Gadiformes	Moridae		
					*Mora moro* (Risso, 1810)	
						Unidentified Moridae
				Macrouridae		Unidentified Macrouridae 1
						Unidentified Macrouridae 2

The thriving coral garden is delimited on its lower part by a slump scar with a visually estimated thickness of a few tens of cm and displaced blocks interpreted as mass wasting evidence. By removing a surficial stratum of the hill flank, this event took away the overlying fauna. No *Crypthelia* was observed within the slump scar. Above the scar’s headscarp, the *Crypthelia* facies is present in what seems to be climatic abundance (Fig. 3a-–g). This sharp transition suggests that previous to the slump the *Crypthelia* garden has likely occupied a broader vertical extent which has since been truncated on its lower part.

The upper limit of the facies, consisting of a transition to a facies dominated by *Narella versluysi* (Fig. 3h) is also relatively sharp but occurs over a few meters, nonetheless.

*Crypthelia vascomarquesi* Zibrowius & Cairns, 1992

### Systematics

**Hydrozoa Owen, 1843**

**Anthoathecata Cornelius, 1992** 

**Stylasteridae Gray, 1847**

***Crypthelia*** **Milne Edwards & Haime,** 1849

**Fig. 4:**
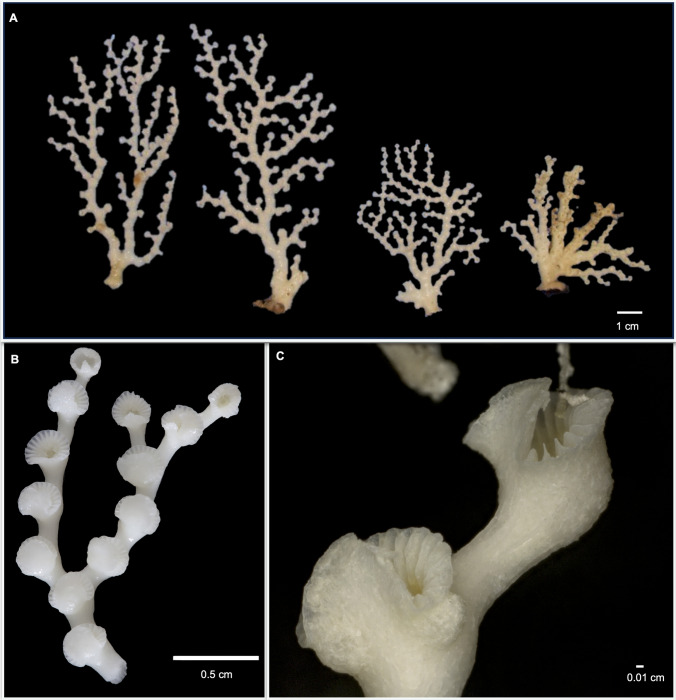
*Crypthelia vascomarquesi* from Menez Gwen Marine Protected Area; **A** Colonies sampled structuring a coral garden; **B** Anterior fragment with 13 cyclosystems, and; **C** Lateral close-up of fragment with two cyclosystems and ampulla

***Crypthelia vascomarquesi*** **Zibrowius & Cairns, **1992**Figures** 4**and** 5

**Fig. 5:**
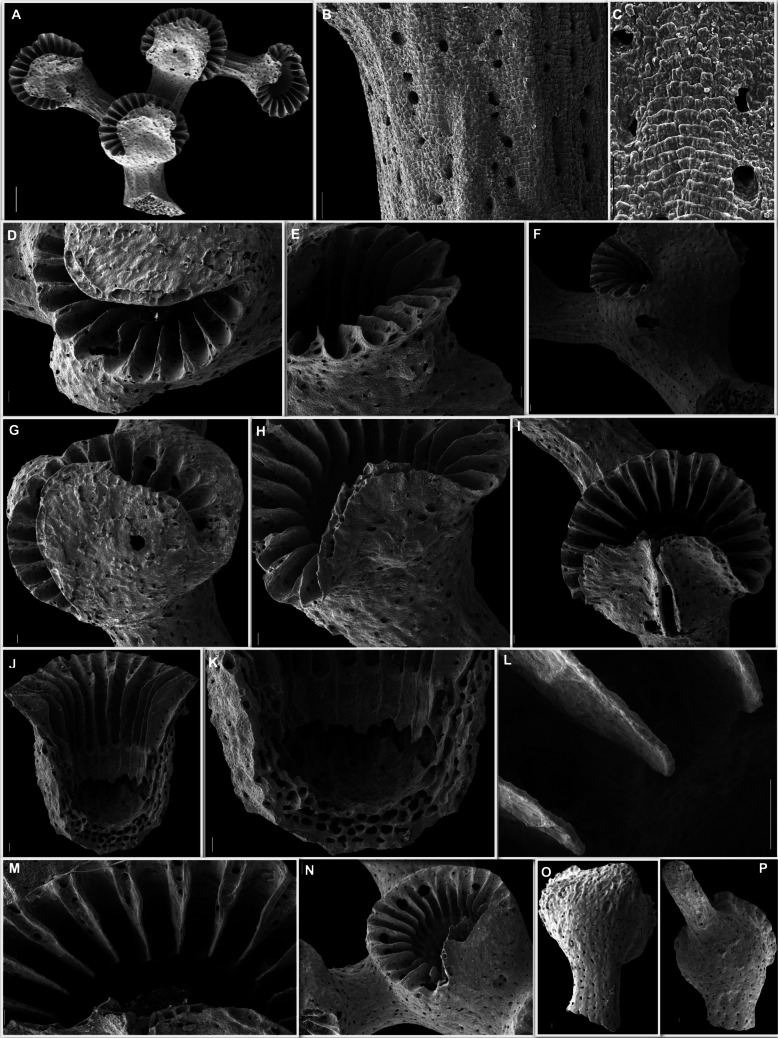
*Crypthelia vascomarquesi* from the Menez Gwen MPA; **A** Distal fragment of colony sampled at the coral garden; **B**–**C** coenosteal texture; **D**–**E** female cyclosystems with ampullae; **F** side view of male cyclosystem with ampullae, **G**–**I** cyclosystem with ampulla on the lid; **G** efferent pores on dactylostomes; **I** rare cyclosystem with two lids; **J** longitudinally-fractured cyclosystem showing the gastropore chambers; **K** detail of a gastropore lower chamber; **L** Pseudosepta detail; **M** nematopores on pseudosepta getting thinner towards the gastropore chamber; **N** cyclosystem with big efferent pores opened in the dactylostomes; **O** anterior view of cyclosystem with male efferent pore, and; **P** anterior view of cyclosystem with female efferent tube. Scale bar: 100 µm

*Crypthelia vascomarquesi* Zibrowius & Cairns, 1992: 114–117, Figs. 39 A-J, 40 A-J.

#### Type Material

 The holotype and two paratypes are deposited at the Muséum national d’Histoire Naturelle (MNHN), Paris (Talisman drag 128). More paratypes sampled during the expedition Biaçores in 1971 at station 232 are also deposited at the MNHN. Other paratype collected during the Prince Albert I of Monaco expeditions, station 242 is deposited in Musée Océanographique de Monaco (MOM), Monaco.

#### Type Locality

Azores, between São Miguel and Faial islands, 16.08.1883, 38^o^07’N, 27^o^11′45’’W, 983 m, “Talisman”, drag 128.

#### Material Studied

Azores, western flank of Menez Gwen Hills, Crypt’Hill, dive 511, biobox 4, 11 colonies and 9 fragments, 832–856 m depth.

#### **Description**

Eleven colonies and nine fragments were used on the redescription of this species totalizing about 590 cyclosystems (Fig. 4, 5a).

Colonies are robust, small and uniplanar harboring from 2 to 97 cyclosystems, while the fragments of subterminal branches harbor from 3 to 27 cyclosystems (Fig. 4). Anastomosis is absent. Eleven colonies range from 0.81 to 6.15 cm length and from 0.27 to 5.88 cm width, presenting a robust calcareous base from which up to four main branches ramify with a maximum thickness of 5.8 mm. Subterminal branches are thinner than the cyclosystems they support (0.78–1.97 mm) (Fig. 4).

The coralla are white in colonies sampled dead or with yellow tissue in colonies sampled alive (Fig. 4). Some colonies were partly dead and partly alive. Coenosteal strips are broad, forming large, straight salient, semi-salient, or not protruding rows in distinct areas of the colonies and more irregular near cyclosystems (Fig. 4c, 5b). Strips can reach up to 290 μm length and 0.22 mm width. The platelets are squared and flat in linear rows, imbricating with normal polarity, and have between 32 and 143 μm width (Fig. 5c).

Cyclosystems are present in the main branches following a straight row until ramification occurs in subterminal branches (Fig. 4). All cyclosystems are positioned on the anterior side of the colonies. Cyclosystems are elliptical with a slightly higher width (2.23–3.49 mm) than length (1.95–3.38 mm). They bulge highly from the branch (Figs. 4 and 5a), sometimes incorporating ampullae on the cyclosystem wall (Fig. 5d-–f).

Gastropores have a maximum upper chamber diameter of 1.34 mm. Cyclosystem lid, localized horizontally on top of the gastropore, is similar to a tongue covering 45–70% of the cyclosystem (Figs. 4 and 5a,g,h). It is straight on the sides and smooth-edged on top, being able to achieve a width of almost 2.27 mm. Two lids were found at a single cyclosystem (Fig. 5i). Gastropores have upper chambers with a maximum diameter of 1.04 mm, while its constriction ring can achieve 1.08 mm (Fig. 5j). The lower chamber has about 0.90 mm of width with a smooth texture (Fig. 5k). After analysis of 539 cyclosystems (out of 590), it was possible to count 316 with a range of 11 to 22 dactylopores per cyclosystem and a mean and mode of 17 dactylopores per cyclosystem. Dactylostomes are wide (0.05–0.38 mm) and the correspondent pseudosepta are thin (0.01–0.15 mm in diameter). Pseudosepta start with wider edges in a skirt-shape and get thinner towards the upper chamber, that curves down with a keel-shape (Fig. 5i-–m). Their edges extend outwards the cyclosystem wall and harbor nematopores. Nematopores are small (17.28–91.47 μm in diameter) and distributed in rows on the edge of the lids of the cyclosystems, randomly distributed in ampullae and coenesteum, or more common and linearly found on the upper and outer pseudosepta (Fig. 5b, c, e, and m).

Male ampullae were located around cyclosystem walls forming a complete ring, or separated bulbs with a diameter of 0.31 to 0.51 mm (Fig. 5f). They have efferent pores with a diameter of 40 to 90 μm, that can be found on top of the ampullae or in dactylostomes. Cyclosystem lids also seem to harbor male ampullae. Female ampullae were located in the coenosteal surface, the cyclosystem wall, and the base or top of cyclosystem lids, having bigger sizes (0.56–1.6 mm) with a maximum presence of 3 per cyclosystem (Fig. 5d, g, h). Respective efferent pores are big (0.10–0.70 mm), opening in the dactylostomes, split half in dactylostomes, and half in pseudosepta or, on top of the ampullae (Fig. 5 d, g, and n). Some male efferent pores were observed (Fig. 5o) and female efferent tubes had 1.30–1.41 mm length (Fig. 5p).

#### Distribution

 Known to occur in the Azores (390–983 m) and Madeira archipelagos (990–1520 m) and the Hyères seamount (600 m). Zibrowius and Cairns (1992) hypothesize that other specimens collected during the CANCAP 3 expedition near Selvagem Grande, Madeira (585 m) and on the Josephine Seamount (622 m) may also belong to this species.

#### Remarks

Although the original description of this species is solid, it was based on colony fragments, lacking representativeness of both female and male colonies (Zibrowius and Cairns 1992). The abundance of specimens collected during this study has allowed to take further measurements of morphological traits and to examine the location of female efferent pores for the first time.

Some morphological features match what had been previously described for *C. vascomarquesi*, simply increasing their distribution range. Cyclosystems diameter has increased to a maximum of 3.5 mm, approaching the maximum value found in *C. tenuiseptata*. Dactylostomes were wider and the area of coverage of the cyclosystem by the lid has also increased considerably from 30–40% to 45–70%. Both diameter range for female and male ampullae and efferent pores are known to be bigger. Double chamber measurements were all near 1 mm like expected while the lower chamber had a smaller diameter than the upper chamber. Nematopore diameter range has decreased from 85 to 17 μm, the lowest nematopore diameter known to all the species of the genus so far. As expected, no anastomosis was found and the number of dactylopores per cyclosystem fell within the expected value for the species.

Nonetheless, some measurements fell within the range known for *C. tenuiseptata*, as is the case of the size of coenosteal strips, despite their organized flat platelets like those found on *C. vascomarquesi*. The length of platelets in the species is now known to be higher. The same is true for the diameter of the lids, revealing a maximum length that went from 1.1 to 2.3 mm in *C. vascomarquesi*.

These specimens were higher and larger than the known specimens of 3 of the 4 species of *Cryptelia* occurring in the Azores. Still, they remained below the known size of *C. tenuiseptata*. Differences between *C. vascomarquesi* and other species occurring in the Azores are the larger size of its cyclosystems, with the exception of *C. tenuiseptata*; the straight and well-defined platelets of the coenosteal texture and the large female efferent pores within the dactylostomes. Its ampullar formula B-C4 + A according to Cairns (1986) or C + C4 in Cairns (2015) is also distinct from the ones known for the other species. Moreover, this is the single species of *Crypthelia* in which bigger female efferent pores are found within the upper area of the dactylostomes, a fact not incorporated in previous ampullar formulas (S. Cairns, *pers. comm.*).

In the eastern Atlantic, this species can be mistakenly identified as *Crypthelia tenuiseptata* Cairns, 1986, as mentioned above, due to its robust colony macromorphology, thin pseudosepta and lid shape. As reported in Zibrowius and Cairns (1992), the species also resembles *Crypthelia glossopoma* Cairns, 1986 from the western Atlantic. Similarities are found on the thin pseudosepta, form of cyclosystem lids, location of nematopores and ampullar formula (Zibrowius and Cairns 1992).

## Discussion

Since the late 1960’s manned submersibles and, more recently, ROVs have collected images of deep-sea fauna within the Azores Exclusive Economic Zone (Pérès 1992). The spatial coverage of these efforts has increased over the last decade in the scope of projects that aimed to locate, describe and map biotopes sensitive to human activities in the deep-sea.

### Biodiversity, taxonomy, and distribution

The latest taxonomic studies of the Azorean stylasterid fauna go back to the early 1990s with the main work being Zibrowius and Cairns (1992). These authors listed nine species of lace corals (Stylasteridae) for the region. The same number was reported roughly two decades later by Braga-Henriques et al. (2013), in a review of the Azorean corals inhabiting the deep sea.

Stylasterids have low species richness in the region, when compared to other groups of corals. Yet, a by-catch analysis highlighted a lace coral, the Azores endemic *Errina dabneyi* (Pourtalès, 1871) as being the second most by-caught coral of long-line fisheries happening between 200 and 500 m of depth (18.6% of total colonies) (Sampaio et al. 2012). The high number of by-caught specimens of *E. dabneyi* may indicate high abundance of the species on the sampling locations selected by fishers, high abundance of the species on the archipelago or a particular selectiveness of the fishing gear for the tallest and most intricate hard corals that colonise the depth range targeted by fishers.

Despite *Crypthelia* being the genus of lace corals with higher species diversity within the Azores, no specimens were recorded in the scope of the by-catch study conducted by Sampaio et al. (2012) in collaboration with professional fishers. Unpublished scientific data indicate that dead tiny fragments of *Crypthelia* were collected in Menez Gwen hydrothermal vent field sediment along with pteropod shells during the cruise EMEPC/LUSO/Açores/2009. Later, during the 2016 MEDWAVES cruise, similar sediment samples were collected on the Formigas bank – another seamount on the easternmost part of the Azorean archipelago. In the same year, larger live colonies were a rare occurrence in the samples collected throughout the archipelago by the M128 scientific cruise. However, the abundance of fragments with cyclosystems in sediments, suggested the presence of living specimens nearby.

During the DEEPFUN cruise in 2012, eleven colonies and nine fragments were sampled within monospecific facies from a single location on the Menez Gwen Hills. These specimens were herein identified and used for re-describing *Crypthelia vascomarquesi*. In this study, we redescribe the species adding details not mentioned before in the original description which was based uniquely in colony fragments. For the first time, *C. vascomarquesi* is described based on entire specimens including female representatives. The redescription increases values of morphological characteristics, formerly thought to be smaller because they were measured in parts of colonies. It is also the first-time efferent pores of the feminine gender are found located on a dactylostome in any species of the genus *Crypthelia*. This detail adds a new category to the feminine ampullar formula developed for this genus by Cairns (1986) and improved by Cairns (2015).

### Habitat and conservation

The herein reported *C. vascomarquesi* facies is the first coral garden formed by a *Crypthelia* species. Until now only fifteen species of stylasterids were known to form deep-sea habitats for other marine invertebrates (Roberts et al. 2009). The occurrence of this species in the Azores matches the general habitat preferences shown by stylasterids. Rather than occurring on continental margins with lower salinity and increased sedimentation, stylasterids prefer small and steep submarine ridges, seamounts and small volcanic oceanic islands with hard substrates and low nutrient levels (Cairns 1992, 2011). Lower levels of competition from other species may also be key to their success in oligotrophic cold-water areas (Cairns 1992).

In the Azores archipelago, this species has the shallowest depth limit of its genus, while inhabiting a depth range that extends below 2000 m. Nonetheless, there is still a considerable lack of knowledge on the taxonomy, distribution, growth, and reproduction of stylasterids in general. We report herein that the volcanic flank colonised by the species has been impacted by an undated mass wasting event and the hard substrates remaining in the slump scar show no evidence of *Crypthelia* recolonization. This may indicate that the species recruitment is episodic and depends on rare favourable circumstances. Other biological features of lace corals may further impede their dispersal: (i) limited distribution ranges, (ii) high endemism, (iii) sexual dimorphism and most notably (iv) the low dispersal ability of the larval stage, with brief planular stage that leads larvae to settle close to the parent colony (Brooke and Stone 2007; Cairns 2011).

On the upper end of the facies, the fast disappearance of *C. vascomarquesi,* over a few meters less than 70 m distance from the summit, is interpreted as resulting from a sharp ecotone possibly related to hydrodynamics. Near-summit turbulence or flow acceleration may therefore represent unfavourable conditions for *C. vascomarquesi*.

Nonetheless, the high-density hard-branched three-dimensional structures documented in the Menez Gwen Hills bring to light *C. vascomarquesi* as a new habitat-engineer that pertains on the list of organisms creating Vulnerable Marine Ecosystems (VMEs). This coral garden is rare as is the species *C. vascomarquesi*, which must now integrate the list of sixteen species of stylasterids known to structure deep-sea habitats. Moreover, several species were found within the surveyed facies, including fishes and other invertebrates (e.g., Cnidaria, Porifera, Crustacea (Table 1)), supporting its structural role. These observations add to Roberts et al. (2009), which point to stylasterids as being providers of physical habitat to marine invertebrates such as Foraminifera, Gastropoda, Ophiuroidea, and Bryozoa. While the MPA regulation is supposed to protect this coral garden from direct anthropogenic impacts, the habitat remains vulnerable to other human-induced stress factors affecting the deep sea, including ocean acidification and deoxygenation, consequences of the climate change. Yet, this rare finding is of importance because it represents a new ecosystem-engineer inhabiting a Marine Protected Area adding to the conservation role of the Menez Gwen MPA, where fisheries are forbidden.

## Conclusions

The first coral garden formed by a *Crypthelia* species is reported in an MPA located within the Azores EEZ. *C. vascomarquesi* forms a monospecific habitat with high density. The new complete specimens analyzed in this study allowed a redescription of this rare species, whose morphology and variation were unrepresented in the original description.

The conservation value of this engineering species is attested by its high density and known endemism level. Therefore, in view of the poor representation of Macaronesian biotopes in the EUNIS habitat classification system, we suggest that this biotope is integrated at EUNIS level 5 in the deep-sea section of this classification under ME123 “Mixed cold water coral communities on Atlantic upper bathyal rock” with the name “*Crypthelia vascomarquesi* gardens on bathyal rock”, in order to support its future documentation and conservation.

## Declarations

### Sampling and field studies

All necessary permits for sampling and observational field studies have been obtained by the authors from the competent authorities and are mentioned in the acknowledgements, if applicable. The study is compliant with CBD and Nagoya protocols.

### Data availability

All the data supporting the findings of this study (e.g. geographical coordinates, morphometric measurements and species list) are available within the paper. Multibeam bathymetry and backscatter datasets are published and cited herein.

## References

[CR1] Borowski C, Meinecke G, Meyer V, Nowald N, Renken J, Ruhland G, Wendt J, Wintersteller P, Thal J (2010) MENEZKART RV POSEIDON No. 402 [POS402], 30.07. – 10.08. 2010, Ponta Delgada – Ponta Delgada (Portugal). Max-Planck-Institut für Marine Mikrobiologie, Bremen, Germany, 32 pp. 10.3289/CR_POS402.

[CR2] Braga-Henriques A, Porteiro FM, Ribeiro PA, De Matos V, Sampaio Í, Ocaña O, Santos RS (2013) Diversity, distribution and spatial structure of the cold-water coral fauna of the Azores (NE Atlantic). Biogeosciences 10(6):4009–4036. 10.5194/bg-10-4009-2013

[CR3] Brooke S, Stone R (2007) Reproduction of deep-water hydrocorals (family Stylasteridae) from the Aleutian Islands, Alaska. Bull Mar Sci 81(3):519–532

[CR4] Cairns SD (1986) A revision of the northwest Atlantic Stylasteridae (Coelenterata: Hydrozoa). Smithsonian Contrib Zool 418:1–131. 10.5479/si.00810282.418

[CR5] Cairns SD (1992) Worldwide distribution of the Stylasteridae (Cnidaria: Hydrozoa). Sci Mar 56:125–130

[CR6] Cairns SD (2011) Global diversity of the Stylasteridae (Cnidaria: Hydrozoa: Athecatae). PLoS One 6(7):e21670. 10.1371/journal.pone.0021670 21799741 10.1371/journal.pone.0021670 PMC3142106

[CR7] Cairns SD (2015) Stylasteridae (Cnidaria: Hydrozoa: Anthoathecata) of the New Caledonian region. Mem Mus Natl Hist Nat 207:1–361

[CR8] Calado H, Ng K, Lopes C, Paramio L (2011) Introducing a legal management instrument for offshore marine protected areas in the Azores—the Azores Marine Park. Environ Sci Policy 14(8):1175–1187. 10.1016/j.envsci.2011.09.001

[CR9] Davies JS, Guillaumont B, Tempera F, Vertino A, Beuck L, Ólafsdóttir SH, Smith C, Fosså JH, Van Den Beld I, Savini A, Rengstorf A, Christophe B, Bourillet J-F, Arnaud-Haond S, Grehan A (2017) A new classification scheme of European cold-water coral habitats: implications for ecosystem-based management of the deep sea. Deep-Sea Res Part II 145:102–109. 10.1016/j.dsr2.2017.04.014

[CR10] Desbruyères D, Biscoito M, Caprais JC, Colaço A, Comtet T, Crassous P, Fouquet Y, Khripounoff A, Le Bris N, Olu K, Riso R, Sarradin P-M, Segonzac M, Vangriesheim A (2001) Variations in deep-sea hydrothermal vent communities on the Mid-Atlantic Ridge near the Azores plateau. Deep Sea Res Part I Oceanogr Res Pap 48(5):1325–1346. 10.1016/S0967-0637(00)00083-2

[CR11] Dubilier N, M82/3 scientific party (2012) Interdisciplinary geological, chemical and biological studies at the Menez Gwen hydrothermal vent field mid-atlantic ridge, at 37º50’N. Meteor-reports NO. 82, Leg 3: 1–73.

[CR12] Dubilier N (2013a) Swath sonar multibeam EM710 bathymetry during METEOR cruise M82/3 with links to raw data files of bathymetry and water column information. Max-Planck-Institut Für Marine Mikrobiologie, PANGAEA Dataset. 10.1594/PANGAEA.819964

[CR13] Dubilier N (2013b) Swath sonar multibeam EM122 bathymetry during METEOR cruise M82/3 with links to raw data files of bathymetry and water column information. Max-Planck-Institut Für Marine Mikrobiologie, PANGAEA Dataset. 10.1594/PANGAEA.819963

[CR14] Duncan PM (1873) A description of the Madreporaria dredged up during the expeditions of hms porcupine in 1869 and 1870. Trans Zool Soc Lond 8(5):303–344. 10.1111/j.1096-3642.1873.tb00560.x

[CR15] Fouquet Y, Charlou JL, Costa I, Donval JP, Radford-Knoery J, Pellé H, Ondréas H, Lourenço N, Segonzac M, Tivey MK (1994) A detailed study of the Lucky-Strike hydrothermal site and discovery of a new hydrothermal site: Menez-Gwen; preliminary results of DIVA 1 cruise (5-29 May, 1994). Inter-Ridge News 3(2):14–17

[CR16] Fouquet Y, Ondréas H, Charlou J-L, Donval JP, Radford-Knoery J, Costa I, Lourenço N, Tivey MK (1995) Atlantic lava lakes and hot vents. Nature 377:201. 10.1038/377201a0

[CR17] Fouquet Y, Eissen J-P, Ondréas H, Barriga F, Batiza R, Danyushevsky L (1998) Extensive volcaniclastic deposits at the Mid-Atlantic Ridge axis: results of deep-water basaltic explosive volcanic activity? Terra Nova 10:280–286. 10.1046/j.1365-3121.1998.00204.x

[CR18] Gomes-Pereira JN, Tempera F (2016) Hydroid gardens of *Nemertesia ramosa* (Lamarck, 1816) in the central North Atlantic. Mar Biodivers 46(1):85–94. 10.1007/s12526-015-0325-9

[CR19] Gray JE (1858) On *Aphrocallistes*, a new genus of Spongiadae from Malacca. Proc Zool Soc Lond 26:114–115. 10.1111/j.1469-7998.1858.tb06352.x

[CR20] Guillaumont B, Carré C, van den Beld I, Beuck L, Davies J (2011) Methodology of annotating videos and still images and development of the software COVER. MESH Atlantic Workshop, Faro, Portugal.

[CR21] Hickson SJ (1909) Alcyonarian and madreporarian corals of the Irish coasts, with description of a new species of *Stachyodes*. Sci Invest Fish Irel 1907(5):1–18

[CR22] Hovland M, Vasshus S, Indreeide A, Austdal L, Nilsen Ø (2002) Mapping and imaging deep-sea coral reefs off Norway, 1982–2000. Hydrobiologia 471(1):13–17. 10.1023/A:1016576514754

[CR23] Johnson JY (1863) Description of a new species of flexible coral belonging to the genus *Juncella*, obtained at Madeira. Proc Zool Soc Lond 1863:505–506

[CR24] Johnson JY (1868) Descriptions of a new genus and a new species of macrurous decapod crustacean belonging to the Penaeidae discovered at Madeira. Proc Zool Soc Lond 1867:895–901

[CR25] Johnson JY (1899) Notes on the Coralliidae of Madeira, with descriptions of two new species. Proc Zool Soc Lond 1899:57–63

[CR26] Jones DO, Arias MB, Audenhaege V et al (2025) Long-term impact and biological recovery in a deep-sea mining track. Nature 642:112–118. 10.1038/s41586-025-08921-3 40139245 10.1038/s41586-025-08921-3 PMC12137123

[CR27] Klischies M, Petersen S, Devey CW (2019) Geological mapping of the Menez Gwen segment at 37°50’N on the Mid-Atlantic Ridge: implications for accretion mechanisms and associated hydrothermal activity at slow-spreading mid-ocean ridges. Mar Geol 412:107–122. 10.1016/j.margeo.2019.03.012

[CR28] Linnaeus L (1758) Systema Naturae per regna tria naturae, secundum classes, ordines, genera, species, cum characteribus, differentiis, synonymis, locis. [The system of nature through the three kingdoms of nature, according to classes, orders, genera, species, with characters, differences, synonyms, places.]. Impensis Direct. Laurentii Salvii. Holmiae, Stockholm

[CR29] de Matos V, Gomes-Pereira JN, Tempera F, Ribeiro PA, Braga-Henriques A, Porteiro F (2014) First record of Antipathella subpinnata (Anthozoa, Antipatharia) in the Azores (NE Atlantic), with description of the first monotypic garden for this species. Deep Sea Res Part II Top Stud Oceanogr 99:113–121. 10.1016/j.dsr2.2013.07.003

[CR30] Marcon Y, Sahling H, Borowski C, dos Santos Ferreira C, Thal J, Bohrmann G (2013) Megafaunal distribution and assessment of total methane and sulfide consumption by mussel beds at Menez Gwen hydrothermal vent, based on geo-referenced photomosaics. Deep Sea Res Part I Oceanogr Res Pap 75:93–109. 10.1016/j.dsr.2013.01.008

[CR31] Marques AFA, Scott SD, Gorton MP, Barriga FJ, Fouquet Y (2009) Pre-eruption history of enriched MORB from the Menez Gwen (37°50′N) and Lucky Strike (37°17′N) hydrothermal systems, Mid-Atlantic Ridge. Lithos 112(1–2):18–39. 10.1016/j.lithos.2009.05.026

[CR32] Milne Edwards H, Haime J (1849) Mémoire sur les Polypes appartenant à la famille des Oculinides, au groupe intermédiaire des Pseudastréides et à la famille des Fongides. CR Acad Sci 29:67–73

[CR33] Milne Edwards A, Bouvier EL (1894) Crustacés décapodes provenant des campagnes du yatch l´Hirondelle (1866, 1887, 1888). Brachyures et Anomoures. Résult Camp Scient Prince Albert I Monaco 7:3–112

[CR34] Morato T, Dominguez-Carrió C, Mohn C, Vicente O, Ramos M, Rodrigues L, Sampaio Í, Taranto GH, Fauconnet L, Tojeira I, Gonçalves EJ, Carreiro-Silva M (2021) Dense cold-water coral garden of *Paragorgia johnsoni* suggests the importance of the Mid-Atlantic Ridge for deep-sea biodiversity. Ecol Evol 11(23):16426–16433. 10.1002/ece3.8319 34938446 10.1002/ece3.8319 PMC8668736

[CR35] Morato T, Juliano M, Pham CK, Carreiro-Silva M, Martins I, Colaço A (2022) Modelling the dispersion of seafloor massive sulphide mining plumes in the Mid Atlantic Ridge around the Azores. Front Mar Sci 9:910940. 10.3389/fmars.2022.910940

[CR36] Moseley HN (1879) On the structure of the Stylasteridae, a family of the hydroid stony corals. Philos Trans R Soc Lond B Biol Sci 169(2):425–503

[CR37] Murray J, Hjort J, Appellöf JJA, Gran HH, Helland-Hansen B (1912) The depths of the ocean: a general account of the modern science of oceanography based largely on the scientific researches of the Norwegian steamer Michael Sars in the North Atlantic, vol 37. Macmillan and Co, London

[CR38] Ondréas H, Fouquet Y, Voisset M, Radford-Knoery J (1997) Detailed study of three contiguous segments of the Mid-Atlantic Ridge, south of the Azores (37ºN to 38º30′ N), using acoustic imaging coupled with submersible observations. Mar Geophys Res 19(3):231–255. 10.1023/A:1004230708943

[CR39] Parson L, Gràcia E, Coller D, German C, Needham D (2000) Second-order segmentation; the relationship between volcanism and tectonism at the MAR, 38 N–35 40′ N. Earth Planet Sci Lett 178(3–4):231–251. 10.1016/S0012-821X(00)00090-X

[CR40] Pérès JM (1992) Le bathyscaphe Francais Archimède aux Acores: Étude bionomique et ecologique du benthos profond. Acoreana Suppl 1992:237–264

[CR41] de Pourtalès LF (1871) Deep-sea corals. Illustrated Catalogue of the Museum of Comparative Zoology at Harvard College. University Press: Welch, Bigelow & Co, Cambridge

[CR42] Prestandrea N (1839) Descrizione di due nuovi Crustacei dei Mari di Messina. Atti Accad gioenia Sci nat Catania XIV(II): 131–136

[CR43] Probert PK, Christiansen S, Gjerde KM, Gubbay S, Santos RS (2007) Management and conservation of seamounts. In: Pitcher TJ, Morato T, Hart PJB, Clark MR, Haggan N, Santos RS (eds) Seamounts: Ecology, Fisheries & Conservation. Blackwell Publishing Ltd., Oxford, pp 442–475

[CR44] Risso A (1810) Ichthyologie de Nice, ou histoire naturelle des poissons du Département des Alpes Maritimes. F. Schoell, Paris. 10.5962/bhl.title.7052

[CR45] Roberts JM, Wheeler A, Freiwald A, Cairns S (2009) Cold-water corals: the biology and geology of deep-sea coral habitats. Cambridge University Press, New York

[CR46] Sampaio Í, Braga-Henriques A, Pham C, Ocaña O, De Matos V, Morato T, Porteiro FM (2012) Cold-water corals landed by bottom longline fisheries in the Azores (north-eastern Atlantic). J Mar Biol Assoc UK 92(7):1547–1555. 10.1017/S0025315412000045

[CR47] Sampaio Í, Freiwald A, Porteiro FM, Carreiro-Silva M (2019) Census of Octocorallia (Cnidaria: Anthozoa) of the Azores (NE Atlantic) with a nomenclature update. Zootaxa 4550(4):451–498. 10.11646/zootaxa.4550.4.1 30790828 10.11646/zootaxa.4550.4.1

[CR48] Santos RS, Colaço A, Christiansen S (2003) Planning the management of deep-sea hydrothermal vent fields MPA in the Azores Triple Junction. Arq Life Mar Sci Supplement 4: xii + 70 pp. http://hdl.handle.net/10400.3/4521

[CR49] Schiellerup H, Ferreira P, González FJ, Marino E, Somoza L, Medialdea T (2021) Deliverable 3.3: metallogeny of hydrothermal deposits in European waters. GeoERA-MINDeSEA project. 66 pp

[CR50] Tempera F, Atchoi E, Amorim P, Gomes-Pereira J, Gonçalves JMS (2013) Atlantic area marine habitats: adding new Macaronesian habitat types from the Azores to the EUNIS habitat classification. MeshAtlantic Technical Report 4(2013):126

[CR51] Wisshak M, López Correa M, Zibrowius H, Jakobsen J, Freiwald A (2009) Skeletal reorganization affects geochemical signals, exemplified in the stylasterid hydrocoral *Errina dabneyi* (Azores Archipelago). Mar Ecol Prog Ser 397:197–208. 10.3354/meps08165

[CR52] Zibrowius H, Cairns SD (1992) Revision of the northeast Atlantic and Mediterranean Stylasteridae (Cnidaria: Hydrozoa). Mém Mus Natl Hist Nat (a, Zool) 153:1–136

